# Incidence of influenza virus-associated Severe Acute Respiratory Infections in three districts in 2018 at Gharbia governorate, Egypt

**DOI:** 10.1371/journal.pgph.0003152

**Published:** 2024-05-03

**Authors:** Hossam Hassan, Amr Kandeel, Manal Fahim, Hanaa Abu ElSood, Shereen Elghazaly, Reham Kamel, Sahar El Shourbagy, Salma Afifi, Mohamed Abdel Fattah, Miyoung Choi, Sang Gyu Lee, Vasuki Rajaguru, Whiejong Han

**Affiliations:** 1 Department of Epidemiology and Surveillance, Preventive Sector, Ministry of Health and Population, Cairo, Egypt; 2 Department of Global Health and Disease Control, Graduate School of Public Health, Yonsei University, Seoul, South Korea; 3 Preventive Sector, Ministry of Health and Population, Cairo, Egypt; 4 Department of Clinical Evidence Research Team, National Evidence-based Healthcare Collaborating Agency, Seoul, South Korea; 5 Department of Preventive Medicine, College of Medicine, Yonsei University, Seoul, South Korea; 6 Department of Healthcare Management, Graduate School of Public Health, Yonsei University, Seoul, South Korea; ICDDR B: International Centre for Diarrhoeal Disease Research Bangladesh, BANGLADESH

## Abstract

**Aims:**

Influenza remains a contributor to substantial global morbidity and mortality. There is very limited data on disease burden in Egypt. The purpose of this study was to estimate the incidence of influenza-associated Severe Acute Respiratory Illness (SARI) in three districts in Gharbia governorate in 2018.

**Methods:**

This study Followed the World Health Organization (WHO) manual for estimating disease burden associated with seasonal influenza. The hospital admission database was screened for SARI patients in three districts at Gharbia governorate in 2018. A hospital admission survey (HAS) was used to define the catchment population. The incidence rate estimation was computed as the number of influenza-positive SARI cases per 100,000 population.

**Results:**

A total of 180 SARI cases were identified in the catchment area. The median age was 23 years [IQR: 2–53], and 45% were males. Out of the total SARI cases, 33.3% influenza was confirmed by the laboratory test of RP-PCR. Influenza A(H3N2) virus predominated representing 55.0% of patients, thanA(H1N1) 26.7% and Flu-B virus 18.3%. Influenza prevailed in winter and spring; no deaths from influenza were reported. The annual incidence of influenza-associated SARIs found higher in <2 years (282 /100,000) and ≥65 years patients (215/100,000) at significant level p<0.001.

**Conclusion:**

The WHO Manual for estimating disease burden associated with seasonal influenza was successfully operationalized in the three districts of Gharbia governorate. It can be used in other districts. A considerable burden was associated with influenza viruses requiring hospitalization, especially among the older adult group.

## Introduction

Globally, influenza viruses can cause substantial mortality during winter months, affecting up to 20% of the population, depending on circulating viruses [1]. Seasonal influenza and respiratory syncytial virus (RSV) are the main contributors to Acute Lower Respiratory Infections (ALRI), and among children with ALRI, influenza is the second most identified pathogen [2]. Burden of the Influenza and related respiratory cases been reported that, the number of seasonal influenza cases ranged from 9 million to 35.6 million and from 140,000 to 710,000 hospitalizations in United states [3], it was estimated about 3.16% of the total outpatient during 2012/2013 in Tunisia [4] and annual positive cases 14% in Lebanon [5]. Influenza has long been regarded as an important disease in the elderly due to its high incidence and associated high risk of hospital admissions and mortality in people over the age of 65years [6]. In a while, research over the last decade has revealed that the infection burden due to hospital admissions for influenza related ALRI in young and very young children is also significant [4–7].

Baseline data describing the burden of seasonal influenza is needed, and there are five groups that are particularly vulnerable to seasonal influenza, either because of their greater exposure risk or because of their greater vulnerability to severe illness [8–10]; healthcare staffs, pregnant women, people with chronic disease, extreme age groups over 65 years and younger than 5 years [7, 11–14]. Most tropical countries, especially in Africa, do not have estimates of the influenza burden [4, 10]. In addition, there are few estimates of influenza burden in the WHO Eastern Mediterranean Region (EMR) [4, 9, 10]. Although several studies have described influenza epidemiology in Egypt [12, 13], data describing seasonal influenza burdens are scarce. To date, only one study ahs been conducted to estimate influenza incidence by determining appropriate population denominators through healthcare utilization surveys (HUS) in an Egyptian district in Lower Egypt [15].

The population of Egypt is estimated to be about 100 million in 2018. Egypt is one of the lower middle-income countries of the EMR with a tropical to temperate climate [8]. During 1999, the Ministry of Health and Population (MoHP) of Egypt has implemented Influenza Like Illness (ILI) sentinel-based influenza surveillance [7–10]. In the wake of the avian influenza A (H5N1) outbreak in 2006, A network of 13 Severe Acute Respiratory Infections (SARI) sentinel sites was established across the country by the MoHP to monitor influenza in hospitals. [8]. In a previous study, it was estimated the incidence of influenza-related hospitalizations as 44 per 100 000 per-son-years with the highest incidence found in the age group 2 to 4 years (166 cases per 100 000-person year: [9, 10]. According to WHO’s Manual for Estimating Disease Burden Associated with Seasonal Influenza, hospital admission survey (HAS) are an alternative method of measuring disease burden [6, 8–11].

Influenza vaccination is one of the important components of the global influenza strategy (2019–2030) launched by WHO-EMR, and annual vaccination is highly recommended for all age groups. The strains were utilized as updated data in the WHO laboratories’ surveillance report. Egypt stated during the 2009 influenza pandemic that the overall number of influenza vaccine doses was 1.37 million doses, covering less than 2% of the total population number, and were expected to be 1.4 million doses during 2017, which was considered very low [8, 12, 13, 15]. Egypt also exhibited the highest of immunization, with coverage 98.1% in the 2011 Hajj season [12]. As, Egypt required pre-departure immunizations for influenza for all pilgrims [15]. Data for vaccine coverage in Egypt is very scarce especially in high-risk groups.

This study aimed to estimate the population in the catchment area and to calculate the incidence of influenza-associated SARI by using the Hospital Admission Survey (HAS) data in three health districts in Gharbia governorate according to the guidelines provided in the WHO manual for disease burden associated with seasonal influenza.

## Material and methods

### Study design and data sources

This study was developed at El Mahallah Fever Hospital (EMFH) in Gharbia governorate, Egypt during the calendar year of 2018 according to the guidelines provided in the WHO manual for estimating disease burden associated with seasonal influenza [3].

To describe the epidemiology of influenza and estimate the annual incidence of influenza-associated hospitalizations we combined information from three data sources: i) the national electronic SARI sentinel surveillance database, ii) the health districts demographic database, and iii) the Hospital Admission Survey (HAS) [8].

We calculated the annual incidence of influenza-associated SARI per 100,000 population using the number of laboratory-confirmed influenza SARI cases admitted to EMFH as the numerator and the catchment population of EMFH as the denominator.


$$\text{Incidence}\;\text{of}\;\text{influenza-associated}\;\text{SARI}=\frac{New\;influenza-associated\;SARI\;cases\;at\;EMFH}{Catchment\;population\;for\;EMFH}$$

**Fig 1:**
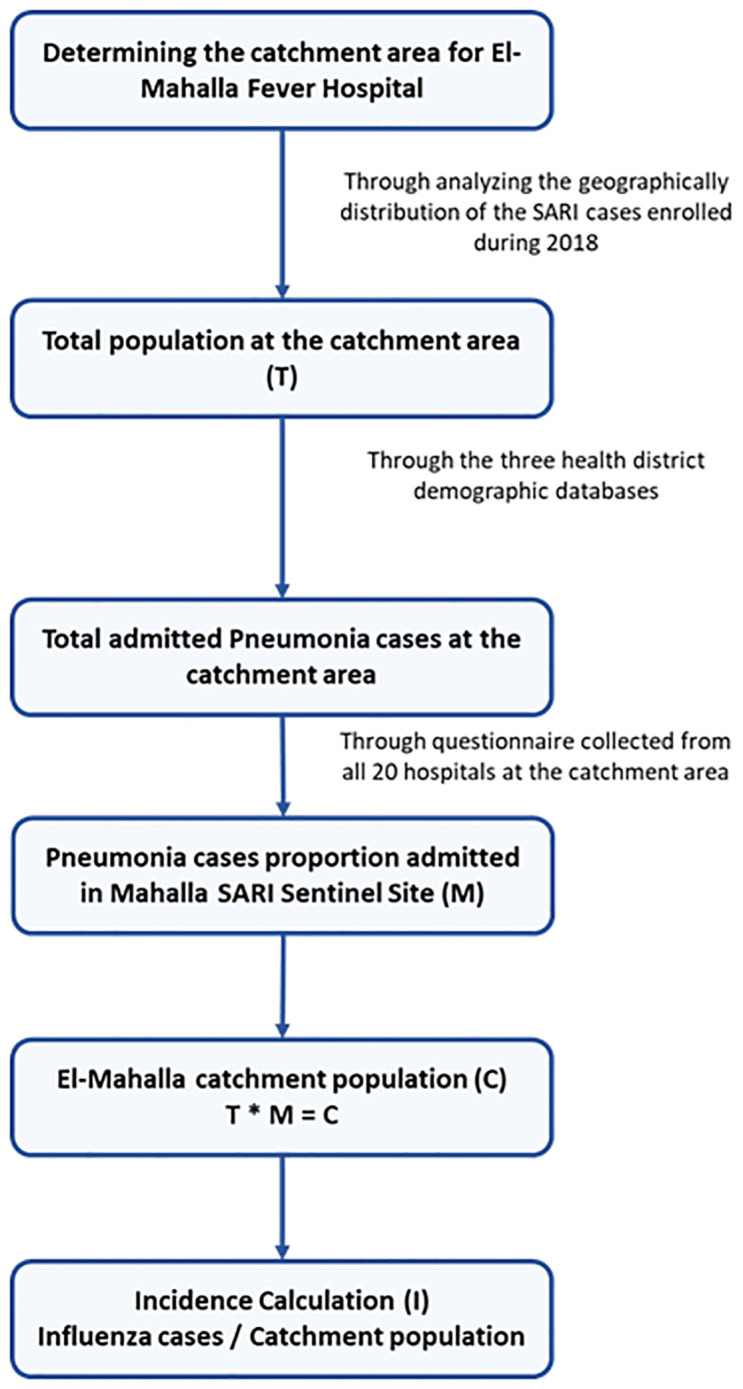
Steps of the incidence estimation algorithm.

The detailed steps of the incidence estimation algorithm are illustrated in Fig 1.

### SARI sentinel surveillance data

Data on influenza-associated SARI enrolled in EMFH from 1 January to 31 December 2018 were extracted from the national electronic SARI sentinel surveillance database.

SARI has been defined as any patient having a history of fever or measured fever of ≥ 100 38°C and cough with onset within the last 10 days and requiring hospitalization [3]. Nasopharyngeal and oropharyngeal swabs were taken from all patients that met standard SARI case definition. Samples were tested for the influenza virus by Real-Time Polymerase Chain Reaction (RT-PCR), influenza types and subtypes (Flu A (H1N1), Flu A (H3N2), and Flu B) were also identified.

### Catchment area determination

**Fig 2:**
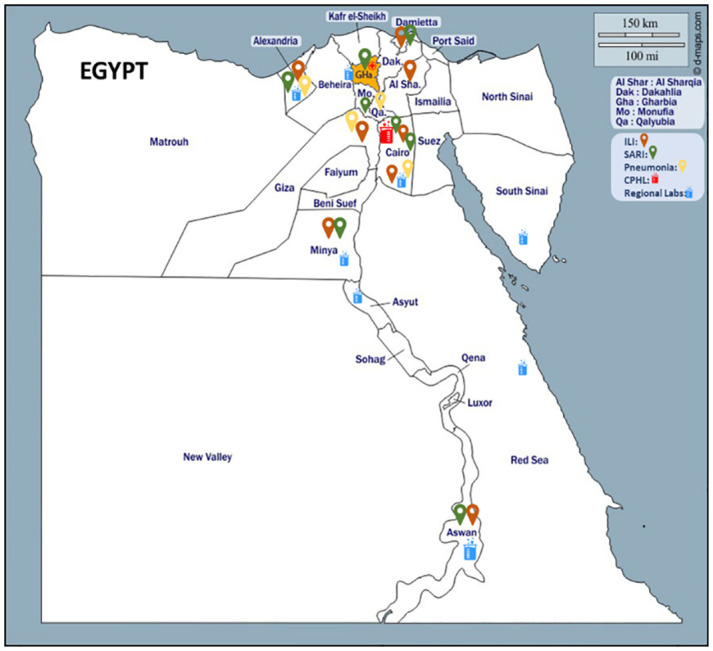
Catchment area map for the enrollment of the SARI cases at Gharbia government.

Amongst the 13 SARI sentinel surveillance sites in Egypt, EMFH serves a well-defined catchment area in Gharbia governorate that was suitable for conducting the HAS. The catchment area was identified according to the WHO manual as the lowest administrative level where the majority of SARI cases (recommended not less than 80%) resided. The catchment area was determined by spotting a map (Fig 2) of all SARI cases enrolled in EMFH during 2018.

Data on the population living in the catchment area was obtained from the health district demographic database. These districts in Gharbia governorate have an estimated population count every year by age group, sex, and residency.

### Counts of pneumonia hospitalizations within the catchment area

#### Hospital admission survey data

To determine the catchment population of EMFH, we developed a cross-sectional HAS. All hospitals located in the catchment area (7 governmental hospitals and 13 private hospitals) were surveyed by a structured questionnaire. For the purpose of HAS we considered pneumonia hospitalizations as a proxy to reflect on SARI hospitalizations, as suggested by the WHO manual [3]. Due to the unified case definition of pneumonia used across all hospitals in the catchment area, including those non-participating in SARI surveillance, calculating pneumonia hospitalization was more feasible [6].

Researchers retrospectively reviewed the admission and discharge records from all hospitals located in the catchment area and counted the number of patients who were hospitalized with pneumonia at each hospital from 1^st^ January to 31^st^ December 2018.

Counts of pneumonia hospitalizations were used to estimate the proportion of respiratory admissions due to pneumonia at the EMFH sentinel site out of all pneumonia admissions at all hospitals in the catchment area. This proportion was further multiplied by population counts retrieved from the health district demographic database to determine EMFH catchment population.

### Data analysis

This study identified and evaluated data sources, as well as checked their accuracy and relevance, in accordance with the WHO manual for seasonal influenza disease burden [3]. Demographic characteristics of influenza and other SARI cases were analyzed using counts and proportions. According to the WHO manual, annual incidence rates and 95% confidence intervals were calculated overall and stratified by age group, sex, and urbanization. Differences in incidence rates between population subgroups were assessed using chi-square test with Epi Info software. Other analyses were conducted in Microsoft excel and Jamovi 2.2.5.

### Ethical approval

The specified data were accessed between 2022.11.11 and 2022.11.20 following ethical committee approval. The study was conducted in accordance with the Declaration of Helsinki and approved by the Egyptian MoHP Research Ethics Committee (REC) under No 18-2022/17. Verbal consent for participation was obtained in accordance with the national legislation and the institutional requirements.

## Results

### General characteristics

**Fig 3:**
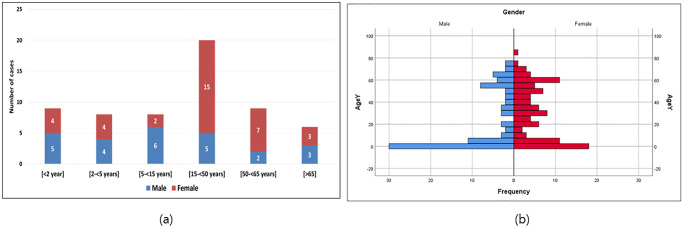
Distribution of El-Mahalla fever hospital catchment population by age group and sex during 2018 and influenza viruses associated Severe Acute Respiratory illness (SARI) by age groups in El-Mahalla fever hospital during 2018.

From 1 January to 31 December 2018, physicians enrolled 216 hospitalized patients who met standard SARI case definition in EMFH. Nasopharyngeal and oropharyngeal swabs were collected from 215 (99.5%). A total of 73 (34%) specimens collected from these patients of all ages were tested positive for influenza virus by RT-PCR. Out of the total SARI cases enrolled at the EMFH, there were 180 SARI cases residing in the catchment area, of which 60 tested positive for influenza. Fig 3 shows the distribution of the population pyramid of the SARI cases (n = 180) by gender, and the distribution of influenza-positive associated SARI by age groups and age (n = 60). In total, 45.6% (82/180) of SARI cases were male, 65.6% (118/180) were rural, 6% (11/180) were chronic diseases, and 0.6% (1/180) were pregnant. There were no deaths or cases admitted to the intensive care unit at EMFH.

### Influenza seasonality

**Fig 4:**
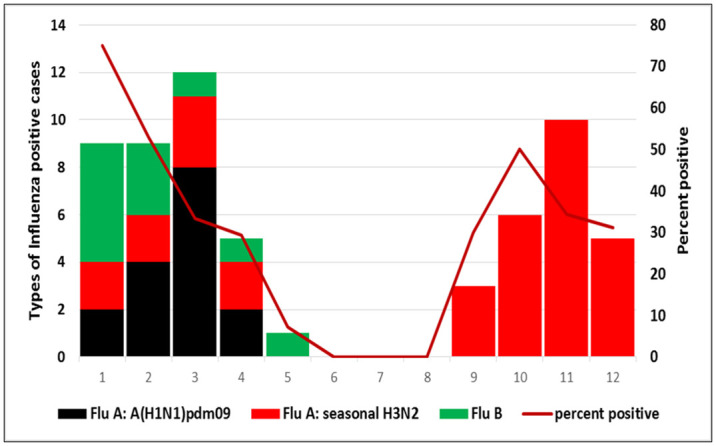
Laboratory-confirmed influenza SARI cases at El-Mahalla fever hospital by months during 2018.

Most cases of influenza in SARI patients occurred in autumn (September: December) and winter (December: March) seasons. The percentage positive of influenza virus associated with SARI cases ranged from 0% during summer months (June~September) and highest cases of influenza such as A(H3N2) virus and influenza A(H1N1) viruses were more commonly reported in winter (January) (Fig 4).

### Population data

The total population in the catchment area during 2018 was 1,792,630. Males were 51.6% and females were 48.4%. Data were collected from the three health districts distributed by gender and age groups as the three districts represented the majority of SARI cases admitted to EMFH, as recommended by WHO manual. The estimated catchment population for EMFH was 190,096 inhabitants, males were 43.4% and females were 56.6%. The estimated population residence in urban areas was 32.8% and the estimated population resided in rural areas was 67.2%.

### Incidence of influenza-associated hospitalizations

According to HAS, this study estimated the total Pneumonia cases at 877 at the catchment area, the proportion of people admitted to EMFH was 10.6%. The estimated catchment population was 190,096 during 2018 for EMFH, and the estimated total number of influenza cases were 765 at the catchment area (Table 1).

**Table 1:** Detailed calculations incidence rates (per 100,000) of influenza virus-associated severe acute respiratory infection by age group at El-Mahala fever hospital, Egypt, 2018.

Indicators*	Age (Y)	0<2		2<5		5<15		15<50		50<65		>65		Total
	Gender	F	M	F	M	F	M	F	M	F	M	F	M	
Total population		41,645	44,400	79,821	85,099	175,263	186,849	426,875	455,100	110,190	117,474	33,838	36,076	1,792,630
Total admitted Pneumonia cases		111	159	23	49	34	31	80	75	80	76	72	78	877
Pneumonia cases		2	8	7	9	4	5	17	6	17	12	4	2	93
Proportion of admitted patients (%)		1.8	5	21.88	18.37	11.76	16.13	21.25	8	21.25	15.79	5.56	2.56	10.6
Catchment Population		750	2234	17,461	15,630	20,619	30,137	90,711	36408	23415	18,549	1880	925	190,096
Influenza cases		4	5	4	4	2	6	15	5	7	2	3	3	60
Overall incidence rate/100000 population		533	224	23	26	10	20	17	14	30	11	160	324	32

Y, Years; F, Female; M, Male;

* at Mahalla SARI Sentinel Site

This study identified 765 influenza-associated SARI cases during 2018 in the catchment area of Mahalla Fever Hospital. The overall estimated annual incidence rate of influenza-associated was 32 (95% CI, 25–41) per 100,000 population (Table 2). The highest incidence 282 (95% CI, 147–542) cases per 100, 000 population was obtained for the age group less than 2 years old followed by the age group more than 65 years, with incidence estimate of 215 (95% CI, 97–479) per 100,000 population. Incidence of influenza-associated hospitalizations estimates were similar in males 30 (95% CI, 20–44) and females 32 (95% CI, 23–45), as well as between people resided at urban areas 34 (95% CI, 6 22–52) and who resided at rural areas 31 (95% CI, 23–42) (Table 2).

**Table 2:** Incidence rates (per 100,000) of influenza virus-associated severe acute respiratory infection by demographic characteristics at El-Mahala fever hospital, Egypt, 2018.

Characteristics		Estimated population of the Sentinel Site	No. positive influenza	Incidence/ 100,000	(95% CI)	p
Age Groups (Years)	0 < 2	3187	9	282	147–542	<0.001
	2 < 5	32,577	8	25	13–50	
	5 < 15	50,139	8	16	8–32	
	15 < 50	130,874	20	15	10–23	
	50 < 65	42,322	9	21	11–40	
	> 65	2797	6	215	97–479	<0.001
Gender	Male	83,013	25	30	20–44	
	Female	108,189	35	32	23–45	0.714
Residence	Urban	62,313	21	34	22–52	0.691
	Rural	127,787	39	31	23–42	
**Total**		190,096	60	32	25–41	

## Discussion

Globally, seasonal influenza is one of the most common respiratory infections. Several factors influence the severity and burden of seasonal influenza, especially the virus type and subtype. During 2018, influenza viruses were associated with a substantial burden of severe illness requiring hospitalization especially among toddlers and older adults at EMFH. In addition, there were no deaths among all SARI associated influenza in comparison to other SARI sentinel sites in Egypt. Vaccination is considered one of the effective tools to decrease the total burden of seasonal influenza and its complications. In Egypt, seasonal influenza vaccination coverage is considered very low, Al-Awaidy and colleagues estimated the overall influenza vaccination coverage in Egypt during 2018 to be less than 2% of the total population [8].

Estimating the incidence of influenza-associated SARI was one of the main results of our study. The numerator was obtained from the SARI sentinel surveillance site at EMFH, and the denominator was estimated through the HAS at the catchment area of our hospital. Regular estimates of incidence are needed to provide policymakers with the overall burden of influenza disease on the population, as there are many factors that influence the severity of seasonal influenza over time. Determining the catchment populations for EMFH was a challenging process, but after the application of the HAS methods as described in the WHO manual, the catchment population can be easily estimated regularly (every year) for other seasonal influenza sentinel surveillance sites, so that a regular denominator can be used to calculate the seasonal influenza incidence for each hospital [15–20].

This study estimated the incidence of influenza-associated SARI 32/100,000 population (95% CI, 25–41), which was comparable to other studies in low and middle-income countries such as Cambodia [11], Ghana [16], Kenya [17], Indonesia [19], South africa [21], Greece [22], Lebanon [23] and south east Asia [24]; which employed the same methods for determining the catchment population and positive hospitalized influenza cases. Our results were higher in comparison to other studies that estimated the incidence of influenza associated 13% SARI per person and the highest incidence was higher at the age group older than 65 years [9, 14–17, 20–22]. A range of factors may explain differences in influenza rates, such as case definition, estimation methods, seasonality of influenza, circulation of different influenza subtypes, and differences in care seeking behaviors. In addition, SARI only represents half of all respiratory admissions [19].

Our findings of influenza incidence were very close to estimates from Ghana, as they estimated the incidence per 100,000 population in medically attended influenza cases were 30 per 100 000 population (95% CI, 13–84), and the highest incidence was found at the age group 0 to 4 years 135 per 100 000 population (95% CI, 120–152) during 2013 to 2015 [10]. Also, our estimates were higher than colleagues from Kenya, who estimated the influenza incidence among SARI to be 21 per 100 000 population (95% CI, 19–23) during 2012 to 2014 [17], and an Oman study also estimated the influenza incidence among hospitalizations to be 0.5 to 15.4 per 100 000 population between 2008 and 2013, and 7.3 to 27.5 per 100 000 population from 2012 to 2015 [18]. The HAS methods were also applied by researchers from Indonesia, who estimated the incidence to be 13 to 19 per 100 000 population during 2013–2016 and the highest incidence was estimated 87 to 114 per 100 000 population at the age group 0 to 4 years [19].

The seasonality of influenza differs according to geographical location, especially in the northern or southern hemisphere. Egypt is located in the northern hemisphere, and the seasonality of influenza occurs mostly during the winter months (epi-week 42 to epi-week 12). The severity of influenza seasons depends greatly on the influenza type and subtype. This study found that Flu A (H3N2) predominated the 2018 season by 55%, followed by Flu A (H1N1) by 26.7%, then Flu B by 18.3%, and influenza prevailed in winter and spring from epi-week 38 until epi-week 18. In comparison to the study, which was conducted in Egypt during 2013, influenza virus type A subtype (H3N2) prevailed by 54%, followed by Flu B virus by 29.3%, then Flu A (H1N1), begin in winter and continued till the end of the spring [9]. It could assist public health authorities in introducing influenza vaccine for high-risk populations, including children younger than 5 years old and the elderly. Further studies are needed to further estimate burden in elderly populations in Egypt.

### Limitations

The study had a few limitations. First, there was a shortage of quality and completeness in the data for some variables, such as symptoms, residency, treatments, and vaccination status. Second, it was the modest capacity and limited resources of EMFH in certain hospital departments, such as the pediatric department and the absence of an intensive care unit that resulted in bias in admitting cases to the hospital. Third, the estimation of the overall burden is constrained by the incidence rate computations. However, we determined the incidence in our study based on only the national influenza SARI sentinel surveillance system. [25–27]. Furthermore, all hospitals within the catchment area did not have any kind of electronic medical records, so we were unable to collect the data smoothly during the HAS. Moreover, this study calculated the incidence of influenza associated SARI for only one year -2018, as a result, influenza virus-associated SARI cases might have been underestimated or overestimated.

## Conclusion

This study focused on the WHO Manual and successfully implemented in Gharbia governorate, Egypt. Influenza-associated SARI incidence rates would be estimated at the national level. A significant burden of influenza hospitalizations is shown by the incidence rates of influenza-associated SARI, particularly in older persons 65 years of age and younger than 2 years. These results suggest that seasonal influenza vaccination may have a major effect on these age groups. Furthermore, influenza preventive, control, and treatment programs can be reinforced by utilizing surveillance data and allocating resources accordingly. Our study findings suggest that expanding population-based surveillance by adding additional influenza sentinel hospitals (ILI and SARI sentinel sites) in order to better understand influenza burden and seasonality. Further research focusing on comorbidities and vaccination and factors associated with SARI cases by utilizing surveillance data, would be required for a more accurate estimate of influenza burdens in the country. An estimation of these findings could provide the baseline information to policy makers regarding the real burden of the seasonal influenza and prioritizing actions needed to combat future influenza pandemics.

### Study implications

Additionally, the study suggests revising national standards and policies for early detection and successful treatment, particularly for young children and those over 65 years. A national influenza vaccine policy is required to reduce the overall burden of seasonal influenza disease, increase preparedness for influenza epidemics and pandemics, strengthen the national health system, and increase vaccination coverage among high-risk groups such as healthcare workers, pregnant women, people with chronic diseases, and people in their elderly years.

## Supporting information

S1 Checklist (DOCX)
